# Ongoing mumps outbreak among adolescents and young adults, Ireland, August 2018 to January 2020

**DOI:** 10.2807/1560-7917.ES.2020.25.4.2000047

**Published:** 2020-01-30

**Authors:** Annamaria Ferenczi, Sarah Gee, Suzanne Cotter, Kevin Kelleher, on behalf of the Mumps Outbreak Control Team

**Affiliations:** 1 Health Service Executive (HSE) - Health Protection Surveillance Centre (HPSC), Dublin, Ireland; 2 European Programme for Intervention Epidemiology Training (EPIET), European Centre for Disease Prevention and Control (ECDC), Stockholm, Sweden; 3 Health Service Executive (HSE) - Public Health and Child Health, Strategic Planning and Transformation, Dublin, Ireland; 4 The study collaborators are acknowledged at the end of the article

**Keywords:** mumps, Ireland, outbreak, young adults, MMR

## Abstract

Between 18 August 2018 and 24 January 2020, 3,736 mumps cases were notified in Ireland. The highest numbers of notifications were observed in the age group 15–24 years. Vaccination status was reported for 32% (n = 1,199) of cases: 72% of these had received two doses of measles-mumps-rubella (MMR) vaccine. Vaccination uptake after free MMR vaccination targeting colleges and universities since early 2019 was low. Therefore, a national media campaign began in January 2020.

A number of discrete mumps outbreaks were reported in Ireland between August and December 2018, but limited to three Health Service Executive (HSE) public health surveillance regions (Western, North-Western and North-Eastern) (n = 372 cases). In 2019, all eight HSE regions were affected (n = 2,762 cases). In January 2020, the outbreak is still ongoing in all regions (n = 602).

Mumps has been a notifiable disease in Ireland since 1988 [1]; and for surveillance, an Irish case definition is used [2]. Enhanced surveillance data, such as vaccination status, clinical complications, and epidemiological links, are collected if local public health resources permit. Data presented in this paper were extracted from the Computerised Infectious Disease Reporting System (CIDR) on 27 January 2020 and are provisional.

Here we report the emergence of the mumps outbreak and population groups affected.

## Description of the outbreak

**Figure 1:**
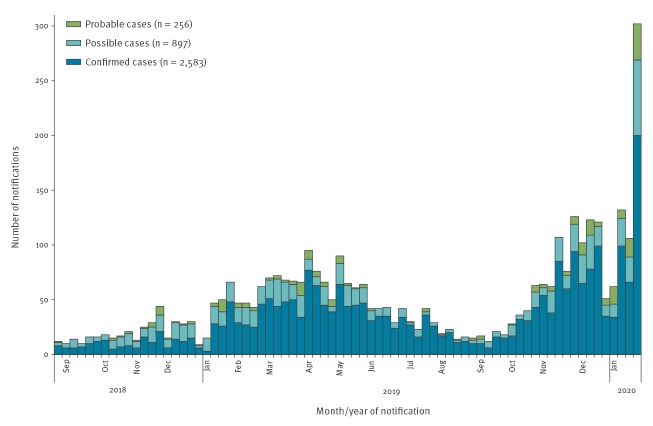
Number of weekly mumps notifications, Ireland, 18 August 2018 (week 34)–24 January 2020 (week 4) (n = 3,736)

In total, between 18 August 2018 and 24 January 2020, 3,736 mumps cases were notified, including confirmed (n = 2,583), possible (n = 897) and probable (n = 256) cases (Figure 1). Mumps activity, which had declined during the summer months of 2019, increased with the start of the academic year in September 2019, and is continuing to increase through January 2020 (Figure 1). The largest number of mumps notifications (n = 302) was reported on week 4 of 2020.

**Figure 2:**
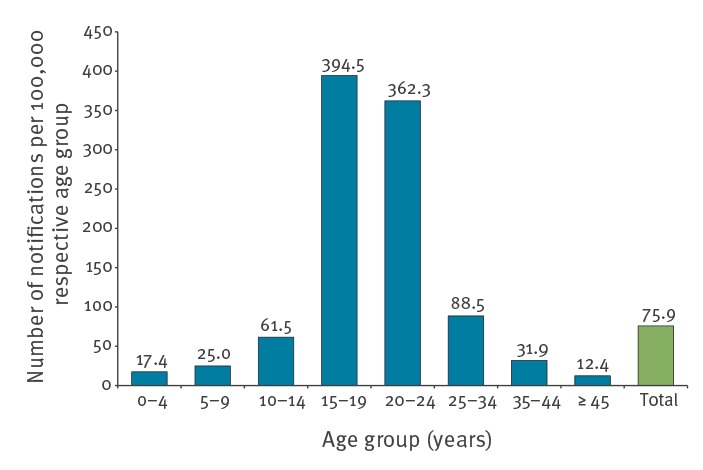
Age-specific incidence rates of mumps notifications, Ireland, 18 August 2018–24 January 2020 (n = 3,728)^a^

^a^ Of the 3,736 cases, age was not reported for eight. Of the 3,728 cases with age reported, 2,581 were confirmed, 893 were possible and 254 were probable.

The highest number of notifications and the highest incidence rates were observed in the age groups 15–19 years and 20–24 years (Figure 2). The median age of the cases was 20 years (interquartile range (IQR): 18–26). Of the cases, 53% were male (1,964/3,736).

The setting in which cases self-reported they were most likely to have acquired mumps was completed for 20% of the notifications (752/3,736). For the majority of these, college/university (44%; n = 333), social setting (20%; n = 147) and secondary school (16%; n = 117) were reported.

Of all cases, 40% (1,505/3,736) were notified in the Dublin region (Eastern surveillance region), most of them from Dublin County (34%; 1,264/3,736).

The National Virus Reference Laboratory (NVRL) genotyped a selection of Mumps RNA positive samples (n = 38) between April 2018 and January 2019, identifying mumps genotype G as the circulating strain.

## Irish measles-mumps-rubella vaccination programme and impact on mumps incidence

**Figure 3:**
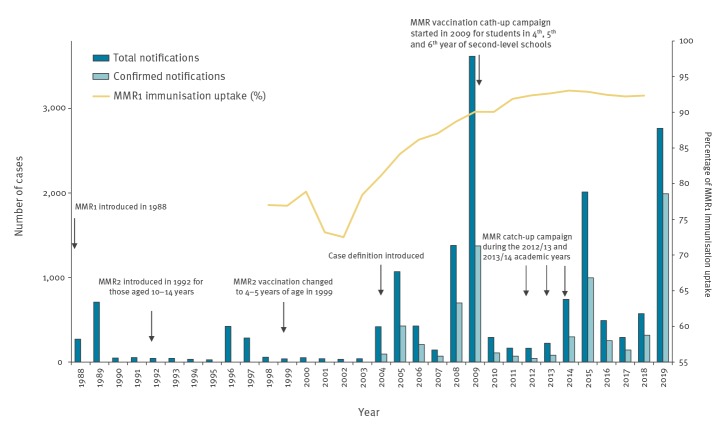
Mumps notifications by year, MMR1 vaccination uptake and history of MMR vaccination, Ireland, 1988–2019

Two doses of the measles-mumps-rubella (MMR) vaccine are estimated to be 88% effective for mumps [3]. Between 1992 and 1998 in Ireland, the second dose of the MMR vaccination was recommended at 10 to 14 years of age (Figure 3). In 1999, the age for the second MMR dose was lowered to address ongoing measles outbreaks among young children. Since 1999, vaccination with the first dose of the MMR vaccine containing the Jeryl Lynn mumps strain has been routinely recommended at 12 months of age and vaccination with the second dose at 4 to 5 years of age (Figure 3).

MMR: measles-mumps-rubella; MMR1: first dose of the MMR vaccination; MMR2: second dose of the MMR vaccination.

Prior to 2004, case classification was not assigned to mumps notifications, therefore the number of confirmed mumps notifications is not known.

Source: Health Service Executive (HSE) Health Protection Surveillance Centre. Summary Immunisation Uptake Statistics Data at 24 months of age.

Since 2004 in Ireland, large mumps outbreaks have occurred every 4 to 6 years (in 2004/05, 2008/09, 2014/15 and 2018/20), with one in similar in size to the current outbreak, when 1,380 cases were reported in 2008 and 3,620 cases in 2009 (Figure 3) [4]. Since 2009, large MMR vaccination catch up-campaigns were implemented in the schools to address gaps in immunity.

The annual first dose of MMR (MMR1) immunisation uptake rates at 24 months of age (available since 1998) are also shown in Figure 3 [5]. The low vaccine uptake reported in 2001/02 was attributed to a lack of confidence in MMR at that time, which led to some parents refusing or delaying vaccination of their children (Figure 3) [6]. The age groups most affected in the current outbreak are the same birth cohort most affected by low MMR1 vaccination uptakes in the early 2000s. Data on the uptake of the MMR vaccine administered predominantly through the school-based programme, which is likely the second dose of MMR (MMR2) for most children, have been only available since the 2011/12 academic school year and range from 83.8% in 2011/12 to 91.5% in 2015/16 [7].

## Vaccination status of cases

**Figure 4:**
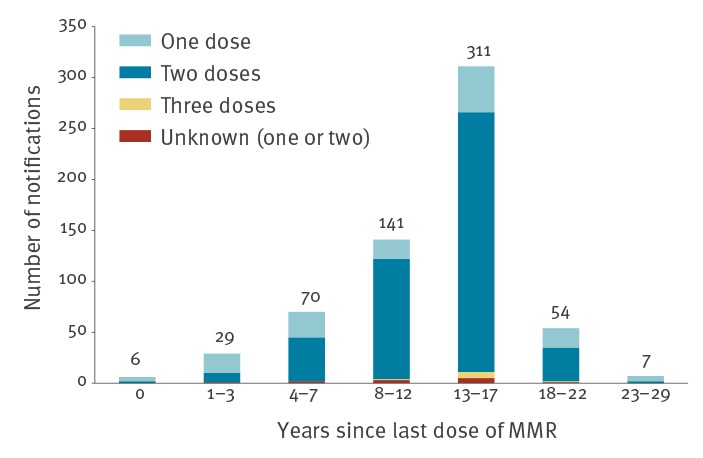
Years between last dose of MMR vaccination received and onset of disease, by doses of MMR, mumps outbreak, Ireland, 2018–2020 (n = 618)^a^

Vaccination status was reported for 32% of cases (1,199/3,736). Where vaccination status was reported, 72% (n = 858) had received two doses of MMR vaccine, 16% (n = 187) had received one dose and 12% (n = 142) of cases were unvaccinated. Twelve cases received a third dose of MMR vaccine. Vaccination information was obtained through a number of sources, such as local immunisation databases, general practitioners’ records, and case/parental records or recall. Because of recall bias, there may therefore be some inaccuracies in the vaccination status figures reported above. We analysed years since the last dose of MMR for cases with reported vaccination date (17%; 618/3,736). Of 618, 52% (n = 323) received the last dose of the vaccination 13 to 17 years before onset of disease (Figure 4).

MMR: measles-mumps-rubella.

^a^ Of 3,736 cases, only 618 had completed information on date of vaccination.

## Severity of illness

Type of patient care was reported for 3,159 cases (85%), of whom 291 (9%) had some form of hospital contact, including treatment at the Accident and Emergency department, day or out-patient care (patients referred as non-emergencies) or hospital admission. Among 137 hospital inpatients, the length of stay (LOS) was reported for 81 cases (60%), with a median LOS of 3 days (IQR: 1–5). Information on any complications was reported for 651 cases, of whom 87 (13%) had some complications. Median age of patients with complications was 20 years (IQR: 18–25). Of those with complications, 79% occurred among males (69/87). Reported complications of mumps included orchitis (5%; 43/855), meningitis (3%; 25/859), pancreatitis (0.5%; 4/839) and encephalitis (0.5%; 4/851). Among those who reported vaccination status, 12% of those with two doses of MMR (99/812) attended hospital compared with 13% of those who were un-vaccinated (18/134).

## Control activities

A national outbreak control team (OCT) was convened in February 2019 to coordinate control and communication efforts throughout the country. The OCT brought together health professionals from all regional public health departments, the NVRL, the Health Service Executive (HSE) National Immunisation Office and the HSE Health Protection Surveillance Centre (HPSC). As the outbreak predominantly affected third-level colleges and universities, students and employees have been targeted for a free MMR vaccination programme advertised through national and local media. Additionally, in 2019, all students (including those in secondary school) and education staff under 25 years of age who had not received two doses of MMR were recommended MMR vaccination. It was also recommended that all new entrants to third-level education younger than 25 years of age be vaccinated with two doses of MMR before the 2019/20 academic year. However, based on most recent data from the HSE drugs payment programme, the number of doses of MMR administered under the code specifically created for outbreaks has been low. Therefore, from January 2020, a media campaign advocates and raises awareness among individuals 11 to 30 years of age across Ireland to ensure they have received two doses of MMR. If vaccination status cannot be confirmed by healthcare or parent record, individuals in this cohort are recommended to get at least one dose of MMR vaccine.

## Discussion

In recent years, many countries, including the United Kingdom (UK) [8], the Netherlands [9], Czech Republic [10], Israel [11] and the United States (US) [12] have reported large mumps outbreaks involving mainly adolescents and young adults. The high potential for transmission in the crowded social environments of students, e.g. large indoor gatherings and shared housing, only partly explains the ongoing outbreak. Other additional factors need to be considered, such as historical low uptake of MMR vaccine (Figure 3), insufficient effectiveness of the mumps component of the MMR vaccine and the possibility of waning immunity in those appropriately immunised. This outbreak again highlights the importance of sustained high vaccination coverage of the two-dose MMR schedule for all children and young adults.

In Ireland, immunisation information systems have traditionally been local, and historically, school immunisations have been held in archived hard copy format and are not easy to routinely obtain. Information on having received MMR vaccination is easier to ascertain, compared to having not received the vaccination. Vaccination status was unknown for 68% of the cases (n=2,537‬) during the ongoing outbreak while only 23% of all cases (n = 858) reported having received two doses of MMR. Such incomplete information on vaccination status of cases made it impossible to determine vaccine effectiveness during the outbreak.

With this rapid communication, we would like to contribute to the discussion about reasons for large mumps outbreaks involving adolescents and young adults occurring in some European countries. As this outbreak is mainly affecting university and college students, which is a highly mobile international population, we would like to raise awareness for the potential of exportation of cases to other countries.

Finally, while students and employees of colleges and universities were targeted for a free MMR vaccination programme since early 2019, the number of vaccinations administered has been low. For this reason, from start of January 2020, we are focusing on raising awareness about mumps in Ireland and the importance of high vaccination coverage of the two-dose MMR vaccination.
